# *tert*-Butyl­ammonium 2,3,4,5-tetra­chloro-6-methoxy­carbonyl­benzoate

**DOI:** 10.1107/S1600536808036398

**Published:** 2008-11-13

**Authors:** Jian Li, Zu-Pei Liang, Cui-Hua Lin, Xi-Shi Tai

**Affiliations:** a Department of Chemistry and Chemical Engineering, Weifang University, Weifang 261061, People’s Republic of China

## Abstract

In the title compound, C_4_H_12_N^+^·C_9_H_3_Cl_4_O_4_^−^, the benzene ring forms dihedral angles of 62.4 (2) and 64.0 (3)°, respectively, with the essentially planar methoxy­carbonyl and carboxyl­ate groups. In the crystal structure, inter­molecular N—H⋯O hydrogen bonds connect anions and cations, forming one-dimensional chains along [010].

## Related literature

For background information, see: Ungwitayatorn *et al.* (2001 ▶). For bond-length data, see: Allen *et al.* (1987 ▶).
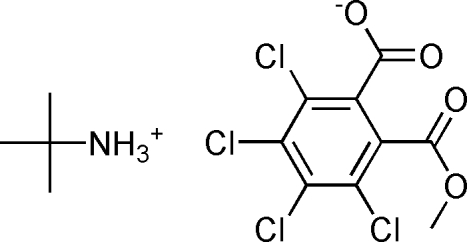


## Experimental

### 

#### Crystal data

C_4_H_12_N^+^·C_9_H_3_Cl_4_O_4_^−^
*M* *_r_* = 391.06
Monoclinic, 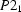

*a* = 9.0193 (14) Å
*b* = 6.5084 (11) Å
*c* = 14.5965 (15) Å
β = 91.7570 (10)°
*V* = 856.4 (2) Å^3^
*Z* = 2
Mo *K*α radiation
μ = 0.70 mm^−1^
*T* = 298 (2) K
0.53 × 0.48 × 0.44 mm

#### Data collection

Bruker SMART CCD diffractometer
Absorption correction: multi-scan (*SADABS*; Sheldrick, 1996 ▶) *T*_min_ = 0.706, *T*_max_ = 0.747
4281 measured reflections
2790 independent reflections
2364 reflections with *I* > 2σ(*I*)
*R*_int_ = 0.043

#### Refinement

*R*[*F* ^2^ > 2σ(*F* ^2^)] = 0.044
*wR*(*F* ^2^) = 0.116
*S* = 1.04
2790 reflections
205 parameters
1 restraint
H-atom parameters constrained
Δρ_max_ = 0.21 e Å^−3^
Δρ_min_ = −0.28 e Å^−3^
Absolute structure: Flack (1983 ▶), 1147 Friedel pairs
Flack parameter: 0.00 (9)

### 

Data collection: *SMART* (Bruker, 2007 ▶); cell refinement: *SAINT* (Bruker, 2007 ▶); data reduction: *SAINT*; program(s) used to solve structure: *SHELXS97* (Sheldrick, 2008 ▶); program(s) used to refine structure: *SHELXL97* (Sheldrick, 2008 ▶); molecular graphics: *SHELXTL* (Sheldrick, 2008 ▶); software used to prepare material for publication: *SHELXTL*.

## Supplementary Material

Crystal structure: contains datablocks global, I. DOI: 10.1107/S1600536808036398/lh2693sup1.cif

Structure factors: contains datablocks I. DOI: 10.1107/S1600536808036398/lh2693Isup2.hkl

Additional supplementary materials:  crystallographic information; 3D view; checkCIF report

## Figures and Tables

**Table 1:** Hydrogen-bond geometry (Å, °)

*D*—H⋯*A*	*D*—H	H⋯*A*	*D*⋯*A*	*D*—H⋯*A*
N1—H1*A*⋯O4^i^	0.89	1.97	2.838 (4)	165
N1—H1*B*⋯O4^ii^	0.89	1.97	2.850 (4)	168
N1—H1*C*⋯O3^iii^	0.89	1.94	2.818 (4)	169

Symmetry codes: (i) 
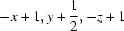
; (ii) 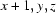; (iii) 
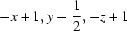
.

**Fig. 1.**
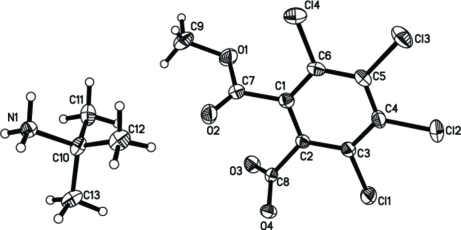
The molecular structure of (I), drawn with 30% probability ellipsoids.

**Fig. 2.**
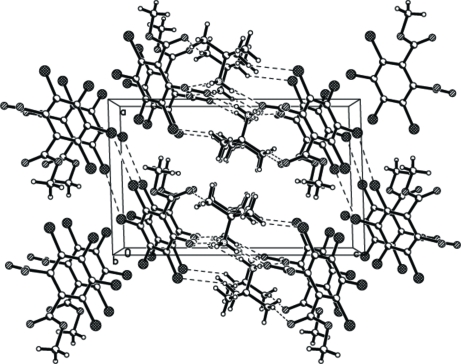
Part of the crystal structure of (I) with hydrogen bonds indicated by dashed lines.

## supplementary crystallographic information

### Comment

Phthalimides are compounds which can posses biological activity (see: e.g. Ungwitayatorn *et al.*,  2001). 2-(Methoxycarbonyl)-3,4,5,6-tetrachlorobenzoic acid is an intermediate in the sysnthesis of tetrachlorophthalimides and their derivatives. In this paper, the structure of the title compound (I) is reported. The asymmetric unit contains one *tert*-butylammonium cation and one 2-(methoxycarbonyl)-3,4,5,6-tetrachlorobenzene-1-carboxylate anion (Fig. 1). The bond lengths in (I) are normal (Allen *et al.*, 1987). In the crystal structure, intermolecular N-H···O hydrogen bonds connect anions and cations to form one-dimensional chains along [O10].

### Experimental

A mixture of tetrachlorophthalic anhydride (2.86 g, 0.01 mol) and methanol (20 ml) was refluxed for 0.5 h and then *tert*-butylamine (0.73 g, 0.01 mol) was added and the mixture stirred for 4 h at room temperature. After filtration, the filtrate was kept at room temperature for 5 d. Natural evaporation gave colourless single crystals of the title compound, suitable for X-ray analysis.

### Refinement

H atoms were initially located from difference maps and then refined in a riding-model approximation with C—H = 0.96 Å, N—H = 0.89 Å and *U*_iso_(H) = 1.5*U*_eq_(N, C).

### Crystal data

**Table:**

C_4_H_12_N^+^·C_9_H_3_Cl_4_O_4_^–^	*F*_000_ = 400
*M**_r_* = 391.06	*D*_x_ = 1.516 Mg m^−^^3^
Monoclinic, *P*2_1_	Mo *K*α radiation λ = 0.71073 Å
Hall symbol: P 2yb	Cell parameters from 2177 reflections
*a* = 9.0193 (14) Å	θ = 2.3–27.0º
*b* = 6.5084 (11) Å	µ = 0.71 mm^−^^1^
*c* = 14.5965 (15) Å	*T* = 298 (2) K
β = 91.7570 (10)º	Block, colorless
*V* = 856.4 (2) Å^3^	0.53 × 0.48 × 0.44 mm
*Z* = 2	

### Data collection

**Table:**

Bruker SMART CCD diffractometer	2790 independent reflections
Radiation source: fine-focus sealed tube	2364 reflections with *I* > 2σ(*I*)
Monochromator: graphite	*R*_int_ = 0.043
*T* = 298(2) K	θ_max_ = 25.0º
φ and ω scans	θ_min_ = 2.3º
Absorption correction: multi-scan(SADABS; Sheldrick, 1996)	*h* = −10→10
*T*_min_ = 0.706, *T*_max_ = 0.747	*k* = −7→7
4281 measured reflections	*l* = −15→17

### Refinement

**Table:**

Refinement on *F*^2^	Hydrogen site location: inferred from neighbouring sites
Least-squares matrix: full	H-atom parameters constrained
*R*[*F*^2^ > 2σ(*F*^2^)] = 0.044	*w* = 1/[σ^2^(*F*_o_^2^) + (0.048*P*)^2^ + 0.2322*P*] where *P* = (*F*_o_^2^ + 2*F*_c_^2^)/3
*wR*(*F*^2^) = 0.116	(Δ/σ)_max_ < 0.001
*S* = 1.04	Δρ_max_ = 0.21 e Å^−^^3^
2790 reflections	Δρ_min_ = −0.28 e Å^−^^3^
205 parameters	Extinction correction: SHELXL97 (Sheldrick, 2008), Fc^*^=kFc[1+0.001xFc^2^λ^3^/sin(2θ)]^-1/4^
1 restraint	Extinction coefficient: 0.075 (5)
Primary atom site location: structure-invariant direct methods	Absolute structure: Flack (1983), 1147 Friedel pairs
Secondary atom site location: difference Fourier map	Flack parameter: 0.00 (9)

### Special details

**Table:**

Geometry. All e.s.d.'s (except the e.s.d. in the dihedral angle between two l.s. planes) are estimated using the full covariance matrix. The cell e.s.d.'s are taken into account individually in the estimation of e.s.d.'s in distances, angles and torsion angles; correlations between e.s.d.'s in cell parameters are only used when they are defined by crystal symmetry. An approximate (isotropic) treatment of cell e.s.d.'s is used for estimating e.s.d.'s involving l.s. planes.
Refinement. Refinement of *F*^2^ against ALL reflections. The weighted *R*-factor *wR* and goodness of fit *S* are based on *F*^2^, conventional *R*-factors *R* are based on *F*, with *F* set to zero for negative *F*^2^. The threshold expression of *F*^2^ > σ(*F*^2^) is used only for calculating *R*-factors(gt) *etc*. and is not relevant to the choice of reflections for refinement. *R*-factors based on *F*^2^ are statistically about twice as large as those based on *F*, and *R*- factors based on ALL data will be even larger.

### Fractional atomic coordinates and isotropic or equivalent isotropic displacement parameters (Å^2^)

**Table:**

	*x*	*y*	*z*	*U*_iso_*/*U*_eq_	
Cl1	−0.17713 (11)	0.5505 (2)	0.24305 (8)	0.0650 (4)	
Cl2	−0.10033 (15)	0.2412 (2)	0.09021 (8)	0.0730 (4)	
Cl3	0.20894 (14)	0.2526 (2)	0.00306 (8)	0.0670 (4)	
Cl4	0.44428 (12)	0.5681 (2)	0.07197 (8)	0.0645 (4)	
N1	0.9054 (3)	0.8116 (5)	0.5564 (2)	0.0332 (7)	
H1A	0.9244	0.9328	0.5825	0.050*	
H1B	0.9627	0.7954	0.5084	0.050*	
H1C	0.9241	0.7119	0.5969	0.050*	
O1	0.4059 (3)	0.9677 (5)	0.1703 (2)	0.0561 (8)	
O2	0.4110 (3)	0.8206 (5)	0.30813 (19)	0.0555 (8)	
O3	0.0729 (3)	1.0097 (4)	0.30549 (18)	0.0462 (7)	
O4	0.0532 (3)	0.7277 (4)	0.39073 (15)	0.0375 (6)	
C1	0.2425 (4)	0.6942 (6)	0.1932 (2)	0.0347 (9)	
C2	0.1041 (4)	0.6869 (6)	0.2345 (2)	0.0323 (8)	
C3	−0.0013 (4)	0.5489 (7)	0.1999 (2)	0.0375 (9)	
C4	0.0307 (5)	0.4133 (7)	0.1295 (3)	0.0431 (10)	
C5	0.1686 (5)	0.4186 (7)	0.0903 (2)	0.0408 (9)	
C6	0.2727 (4)	0.5608 (7)	0.1215 (2)	0.0380 (9)	
C7	0.3626 (4)	0.8340 (6)	0.2319 (3)	0.0378 (9)	
C8	0.0731 (4)	0.8226 (6)	0.3175 (2)	0.0305 (8)	
C9	0.5402 (5)	1.0814 (9)	0.1945 (4)	0.0701 (15)	
H9A	0.6216	0.9874	0.2027	0.105*	
H9B	0.5265	1.1554	0.2505	0.105*	
H9C	0.5614	1.1766	0.1464	0.105*	
C10	0.7440 (4)	0.8028 (6)	0.5251 (3)	0.0367 (9)	
C11	0.7195 (5)	0.9690 (7)	0.4533 (3)	0.0511 (11)	
H11A	0.7391	1.1011	0.4804	0.077*	
H11B	0.6186	0.9642	0.4305	0.077*	
H11C	0.7852	0.9469	0.4038	0.077*	
C12	0.7164 (4)	0.5891 (7)	0.4845 (3)	0.0469 (10)	
H12A	0.7874	0.5622	0.4383	0.070*	
H12B	0.6180	0.5831	0.4576	0.070*	
H12C	0.7264	0.4878	0.5321	0.070*	
C13	0.6520 (4)	0.8364 (7)	0.6089 (3)	0.0487 (11)	
H13A	0.5497	0.8082	0.5938	0.073*	
H13B	0.6622	0.9763	0.6290	0.073*	
H13C	0.6859	0.7459	0.6571	0.073*	

### Atomic displacement parameters (Å^2^)

**Table:**

	*U*^11^	*U*^22^	*U*^33^	*U*^12^	*U*^13^	*U*^23^
Cl1	0.0399 (6)	0.0959 (10)	0.0596 (7)	−0.0201 (6)	0.0101 (5)	−0.0272 (7)
Cl2	0.0733 (8)	0.0769 (10)	0.0685 (8)	−0.0297 (7)	−0.0010 (6)	−0.0322 (7)
Cl3	0.0782 (8)	0.0697 (9)	0.0532 (6)	0.0129 (7)	0.0033 (5)	−0.0277 (7)
Cl4	0.0511 (6)	0.0811 (9)	0.0627 (7)	0.0029 (6)	0.0236 (5)	−0.0087 (7)
N1	0.0333 (15)	0.0302 (17)	0.0364 (16)	0.0004 (13)	0.0033 (12)	0.0002 (14)
O1	0.0603 (19)	0.059 (2)	0.0495 (17)	−0.0197 (15)	0.0029 (14)	0.0158 (15)
O2	0.0606 (18)	0.061 (2)	0.0437 (16)	−0.0225 (16)	−0.0119 (14)	0.0098 (15)
O3	0.0623 (18)	0.0323 (18)	0.0439 (15)	−0.0008 (13)	0.0001 (13)	−0.0013 (12)
O4	0.0446 (14)	0.0374 (15)	0.0308 (13)	0.0003 (13)	0.0076 (11)	−0.0008 (12)
C1	0.039 (2)	0.035 (2)	0.0301 (18)	0.0012 (16)	−0.0010 (15)	0.0053 (16)
C2	0.0373 (19)	0.034 (2)	0.0259 (17)	−0.0030 (16)	0.0004 (15)	0.0015 (15)
C3	0.0349 (19)	0.045 (2)	0.0321 (18)	−0.0021 (19)	0.0002 (14)	−0.0037 (19)
C4	0.050 (2)	0.042 (2)	0.036 (2)	−0.0076 (19)	−0.0054 (18)	−0.0059 (18)
C5	0.050 (2)	0.042 (2)	0.0309 (19)	0.0048 (19)	0.0001 (17)	−0.0057 (17)
C6	0.0403 (19)	0.045 (2)	0.0286 (17)	0.007 (2)	0.0061 (15)	0.0042 (19)
C7	0.036 (2)	0.040 (2)	0.038 (2)	−0.0019 (17)	0.0050 (17)	0.0023 (18)
C8	0.0290 (18)	0.028 (2)	0.0340 (19)	−0.0036 (16)	−0.0012 (15)	0.0000 (16)
C9	0.064 (3)	0.070 (4)	0.078 (3)	−0.032 (3)	0.022 (2)	0.003 (3)
C10	0.0268 (17)	0.033 (2)	0.050 (2)	0.0016 (16)	−0.0027 (15)	−0.0039 (18)
C11	0.045 (2)	0.050 (3)	0.057 (3)	0.007 (2)	−0.011 (2)	0.010 (2)
C12	0.039 (2)	0.042 (3)	0.059 (3)	−0.0065 (19)	−0.0001 (18)	−0.013 (2)
C13	0.040 (2)	0.045 (3)	0.062 (3)	0.006 (2)	0.0158 (19)	−0.007 (2)

### Geometric parameters (Å, °)

**Table:**

Cl1—C3	1.724 (4)	C3—C4	1.392 (6)
Cl2—C4	1.714 (4)	C4—C5	1.385 (6)
Cl3—C5	1.718 (4)	C5—C6	1.385 (6)
Cl4—C6	1.729 (4)	C9—H9A	0.9600
N1—C10	1.513 (5)	C9—H9B	0.9600
N1—H1A	0.8900	C9—H9C	0.9600
N1—H1B	0.8900	C10—C13	1.515 (5)
N1—H1C	0.8900	C10—C11	1.517 (6)
O1—C7	1.319 (5)	C10—C12	1.529 (6)
O1—C9	1.455 (5)	C11—H11A	0.9600
O2—C7	1.186 (4)	C11—H11B	0.9600
O3—C8	1.230 (5)	C11—H11C	0.9600
O4—C8	1.252 (4)	C12—H12A	0.9600
C1—C6	1.394 (5)	C12—H12B	0.9600
C1—C2	1.404 (5)	C12—H12C	0.9600
C1—C7	1.511 (5)	C13—H13A	0.9600
C2—C3	1.391 (5)	C13—H13B	0.9600
C2—C8	1.532 (5)	C13—H13C	0.9600
			
C10—N1—H1A	109.5	O1—C9—H9A	109.5
C10—N1—H1B	109.5	O1—C9—H9B	109.5
H1A—N1—H1B	109.5	H9A—C9—H9B	109.5
C10—N1—H1C	109.5	O1—C9—H9C	109.5
H1A—N1—H1C	109.5	H9A—C9—H9C	109.5
H1B—N1—H1C	109.5	H9B—C9—H9C	109.5
C7—O1—C9	115.6 (3)	N1—C10—C13	107.2 (3)
C6—C1—C2	119.9 (3)	N1—C10—C11	107.5 (3)
C6—C1—C7	120.1 (3)	C13—C10—C11	112.5 (3)
C2—C1—C7	119.7 (3)	N1—C10—C12	107.2 (3)
C3—C2—C1	118.2 (3)	C13—C10—C12	110.9 (4)
C3—C2—C8	121.3 (3)	C11—C10—C12	111.2 (3)
C1—C2—C8	120.5 (3)	C10—C11—H11A	109.5
C2—C3—C4	121.5 (3)	C10—C11—H11B	109.5
C2—C3—Cl1	119.3 (3)	H11A—C11—H11B	109.5
C4—C3—Cl1	119.1 (3)	C10—C11—H11C	109.5
C5—C4—C3	119.8 (4)	H11A—C11—H11C	109.5
C5—C4—Cl2	119.8 (3)	H11B—C11—H11C	109.5
C3—C4—Cl2	120.3 (3)	C10—C12—H12A	109.5
C6—C5—C4	119.3 (4)	C10—C12—H12B	109.5
C6—C5—Cl3	120.4 (3)	H12A—C12—H12B	109.5
C4—C5—Cl3	120.2 (3)	C10—C12—H12C	109.5
C5—C6—C1	121.1 (3)	H12A—C12—H12C	109.5
C5—C6—Cl4	119.2 (3)	H12B—C12—H12C	109.5
C1—C6—Cl4	119.7 (3)	C10—C13—H13A	109.5
O2—C7—O1	125.4 (4)	C10—C13—H13B	109.5
O2—C7—C1	123.1 (4)	H13A—C13—H13B	109.5
O1—C7—C1	111.5 (3)	C10—C13—H13C	109.5
O3—C8—O4	127.6 (3)	H13A—C13—H13C	109.5
O3—C8—C2	117.2 (3)	H13B—C13—H13C	109.5
O4—C8—C2	115.2 (3)		
			
C6—C1—C2—C3	−2.0 (5)	Cl3—C5—C6—C1	−179.0 (3)
C7—C1—C2—C3	−176.8 (3)	C4—C5—C6—Cl4	−179.6 (3)
C6—C1—C2—C8	175.9 (3)	Cl3—C5—C6—Cl4	−0.5 (5)
C7—C1—C2—C8	1.2 (5)	C2—C1—C6—C5	−0.5 (5)
C1—C2—C3—C4	3.3 (6)	C7—C1—C6—C5	174.2 (4)
C8—C2—C3—C4	−174.6 (4)	C2—C1—C6—Cl4	−179.1 (3)
C1—C2—C3—Cl1	−174.8 (3)	C7—C1—C6—Cl4	−4.3 (5)
C8—C2—C3—Cl1	7.3 (5)	C9—O1—C7—O2	11.0 (6)
C2—C3—C4—C5	−2.0 (6)	C9—O1—C7—C1	−168.3 (4)
Cl1—C3—C4—C5	176.1 (3)	C6—C1—C7—O2	−115.3 (4)
C2—C3—C4—Cl2	178.5 (3)	C2—C1—C7—O2	59.4 (5)
Cl1—C3—C4—Cl2	−3.4 (5)	C6—C1—C7—O1	64.0 (5)
C3—C4—C5—C6	−0.6 (6)	C2—C1—C7—O1	−121.3 (4)
Cl2—C4—C5—C6	178.8 (3)	C3—C2—C8—O3	−117.6 (4)
C3—C4—C5—Cl3	−179.8 (3)	C1—C2—C8—O3	64.5 (5)
Cl2—C4—C5—Cl3	−0.3 (5)	C3—C2—C8—O4	63.6 (5)
C4—C5—C6—C1	1.9 (6)	C1—C2—C8—O4	−114.3 (4)

### Hydrogen-bond geometry (Å, °)

**Table:**

*D*—H···*A*	*D*—H	H···*A*	*D*···*A*	*D*—H···*A*
N1—H1A···O4^i^	0.89	1.97	2.838 (4)	165
N1—H1B···O4^ii^	0.89	1.97	2.850 (4)	168
N1—H1C···O3^iii^	0.89	1.94	2.818 (4)	169

Symmetry codes: (i) −*x*+1, *y*+1/2, −*z*+1; (ii) *x*+1, *y*, *z*; (iii) −*x*+1, *y*−1/2, −*z*+1.
